# Beta-lactams susceptibility testing of penicillin-resistant, ampicillin-susceptible *Enterococcus faecalis* isolates: a comparative assessment of Etest and disk diffusion methods against broth dilution

**DOI:** 10.1186/s12941-020-00386-8

**Published:** 2020-09-17

**Authors:** Natália Conceição, Wellington Francisco Rodrigues, Kessys Lorrânya Peralta de Oliveira, Lucas Emanuel Pinheiro da Silva, Laís Rezende Cardoso de Souza, Cristina da de Cunha Hueb Barata Oliveira, Adriana Gonçalves de Oliveira

**Affiliations:** 1 Instituto de Ciências Biológicas e Naturais, Universidade Federal do Triângulo Mineiro, Praça Manoel Terra, 330, 38015-050, Uberaba, Minas Gerais Brazil; 2 Instituto Federal de Educação, Ciência e Tecnologia de Rondônia, Colorado Do Oeste, Rondônia Brazil; 3 Programa de Pós-graduação em Ciências da Saúde, Universidade Federal do Triângulo Mineiro, Uberaba, Minas Gerais Brazil; 4 Instituto de Ciências da Saúde, Universidade Federal do Triângulo Mineiro, Uberaba, Minas Gerais Brazil

**Keywords:** Enterococci, Etest, Disk diffusion, Beta-lactams, Piperacillin, Imipenem

## Abstract

This study aimed to evaluate the accuracy of disk diffusion and Etest methods, compared to that of the broth dilution reference method for identifying beta-lactam susceptibilities of Penicillin-Resistant, Ampicillin-Susceptible *Enterococcus faecalis* (PRASEF) isolates. Fifty-nine PRASEF and 15 Penicillin-Susceptible, Ampicillin-Susceptible *E. faecalis* (PSASEF) clinical nonrepetitive isolates were evaluated. The effectiveness of five beta-lactams (ampicillin, amoxicillin, imipenem, penicillin, and piperacillin) was tested. All antimicrobial susceptibility tests were performed and interpreted according to the Clinical and Laboratory Standards Institute guidelines. Interpretative discrepancies, such as essential agreement, categorical agreement, and errors, were assessed. The acceptability was ≥ 90% for both categorical agreement and essential agreement. Etest proved to be an accurate method for testing beta-lactam susceptibilities of the emerging PRASEF isolates, disk diffusion presented poor performance, particularly for imipenem and piperacillin.

## Background

Although enterococci are widely distributed in the environment and are considered as normal intestinal microbes of humans and animals, during the past few decades, they have caused various infections in humans, primarily observed in hospitalized patients [1]. *E. faecalis,* an enterococci species, is most frequently isolated from clinical specimens [2]. These microorganisms are intrinsically resistant to several antimicrobial agents and have a great ability to acquire and express new resistance determinants. Notably, in recent years, enterococci have acquired high-level antibiotic resistance to aminoglycosides, glycopeptides, and beta-lactams [1, 3].

Enterococci usually present cross-susceptibility to β-lactamase-susceptible penicillin; however, the emergence of clinical penicillin-resistant, ampicillin-susceptible *Enterococcus faecalis* (PRASEF) isolates, exhibiting an unusual resistance phenotype, have been reported in various countries [4–8]. Moreover, although the current Clinical and Laboratory Standards Institute (CLSI) [9] and European Committee on Antimicrobial Susceptibility Testing (EUCAST) [10] guidelines state that the susceptibility to ampicillin may predict susceptibility to amoxicillin, piperacillin*, and imipenem for E. faecalis*, studies have demonstrated that this rule may not be applicable to the penicillin resistant isolates [3, 4, 6, 8].

Various methods are applied for the in vitro evaluation of enterococci susceptibility to beta-lactams; however, disk diffusion and Etest are routinely used in most clinical microbiology laboratories in the developing countries [11, 12]. Thus, this study aimed to assess the performance of Etest and disk diffusion methods to determine the susceptibilities of PRASEF isolates for beta-lactam antimicrobials in comparison with broth dilution using the reference method.

## Methods

### Bacterial isolates and species identification

In total, 59 PRASEF nonrepetitive isolates recovered from patients admitted at a Brazilian tertiary hospital during the period 2006–2014 were included in this study. A few isolates evaluated herein belonged to a previous publication [6, 13]. Moreover, 15 penicillin-susceptible, ampicillin-susceptible *E. faecalis* (PSASEF) isolates were evaluated for comparative testing. The species identification of isolates was based on the phenotypic tests [2] and was confirmed by PCR using specific primers [14]. No isolate produced beta-lactamase according to the results of the nitrocefin disk test (Becton Dickinson and Company).

### Antimicrobial susceptibility testing

Antimicrobial susceptibility testing methods (broth dilution, disk diffusion, and Etest) were run simultaneously, and the standardized inoculum was prepared from the same bacterial suspension. The direct colony suspension method was used for preparing inoculum from colonies grown within 18–24 h at 35 ± 2 °C. Five beta-lactams were tested (penicillin, ampicillin, amoxicillin, imipenem, and piperacillin). Broth dilution and disk diffusion were performed and interpreted according to CLSI guidelines [9]. Etest (bioMérieux, Sweden) was performed according to the manufacturer’s instructions.

The broth dilution method was carried out using cation-adjusted Mueller–Hinton broth (Difco, France) and antimicrobial solutions were prepared from powders of known potencies (Sigma-Aldrich, Denmark). The beta-lactam dilutions tested ranged from 0.5 to 256 µg/mL. *E. faecalis* ATTC 29212 was used as a susceptible control. The same strain was used as a control for Etest assays. A minimum inhibitory concentration (MIC) ≥ 16 µg/mL indicated resistance to all beta-lactams evaluated [9].

Beta-lactam disks (Oxoid, United Kingdom) and Mueller–Hinton agar (Difco, France) were used for disk diffusion testing. *Staphylococcus aureus* ATCC 25923 and *Escherichia coli* ATCC 25922 were included as susceptible controls. An inhibition zone diameter ≥ 15 mm indicated penicillin susceptibility and ≥ 17 mm indicated susceptibility to other beta-lactams [9].

### Data interpretation and analysis

Disk diffusion and Etest results were compared with those obtained by the broth dilution method, which is considered the gold standard. Interpretative discrepancies such as essential agreement (EA; MIC ± 1 log2), categorical agreement (CA; same category result), very major error (VME; false susceptible), and major error (ME; false resistant) were assessed as described elsewhere [11]. The acceptability was ≥ 90% for both CA and EA.

## Results and discussion

**Fig. 1:**
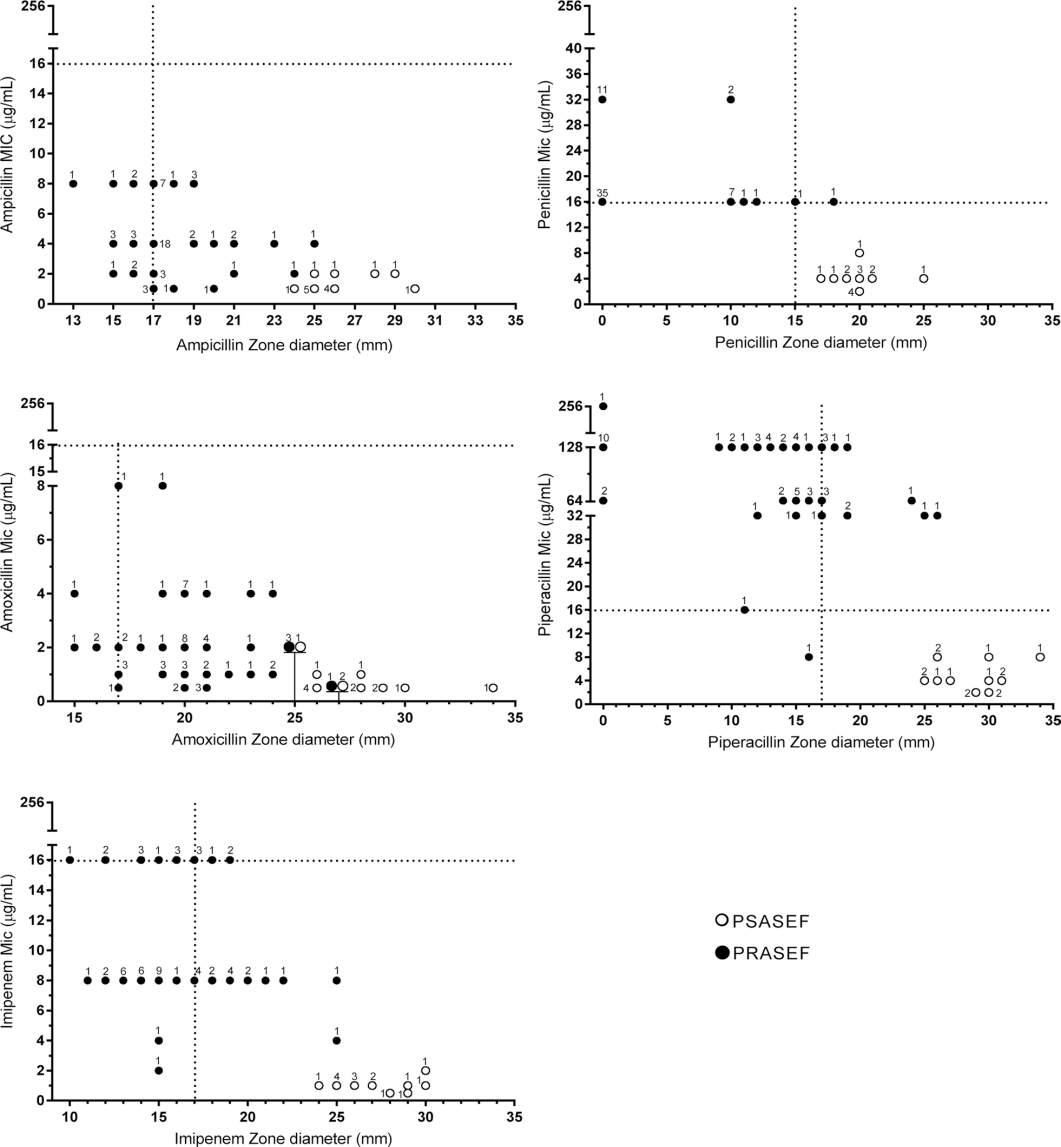
Scattergram of beta-lactam zone diameters of the disk diffusion and minimum inhibitory concentration (MIC) from the reference method of the penicillin-susceptible, ampicillin-susceptible *E. faecalis* (PSASEF; *n* = 15) and penicillin-resistant, ampicillin-susceptible *E. faecalis* (PRASEF; *n* = 59) isolates. Current CLSI breakpoints are represented as dotted lines. Numbers represent the number of isolates at each MIC/zone diameter pair

Table 1 summarizes the results of beta-lactam antimicrobial susceptibility testing of *E. faecalis* isolates and the interpretative discrepancies for each method, whereas Fig. 1 illustrates the relationship of beta-lactam MICs by the reference method and the zone diameters by disk diffusion for PRASEF and PSASEF isolates.

**Table 1:** Beta-lactam susceptibilities of penicillin-resistant, ampicillin-susceptible *Enterococcus faecalis* (*n* = 59) and penicillin-susceptible, ampicillin-susceptible *E. faecalis* (*n* = 15) isolates according to different methods and the interpretative discrepancies considering the broth dilution as gold standard

Antimicrobial	Method	Number of isolates		Number (%) of		EA (%)	CA (%)
		Resistant	Susceptible	VME	ME		
Penicillin	Broth dilution	59	15				
	Etest	54	20	5 (8.5)	0	91.9	93.4
	Disk diffusion	57	17	2 (3.4)	0	NA^e^	97.3
Ampicillin	Broth dilution	0	74				
	Etest	0	74	NA	0	91.9	100
	Disk diffusion	13	61	NA	13 (17.6)	NA^e^	82.4
Amoxicillin	Broth dilution	0	74				
	Etest	0	74	NA	0	91.9	100
	Disk diffusion	4	70	NA	4 (5.4)	NA	94.6
Imipenem	Broth dilution	16	58				
	Etest	27	47	0	11 (19.0)	94.6	85.1
	Disk diffusion	37	37	6 (37.5)	27 (46.5)	NA^e^	55.4
Piperacillin	Broth dilution	58	16				
	Etest	57	17	1 (1.7)	0	75.7	98.6
	Disk diffusion	45	29	14 (24.1)	1 (6.3)	NA^e^	79.7

*VME* very major errors (false susceptibility), *ME* major errors (false resistance), *EA* essential agreement, *CA* categorical agreement, *NA* not applicable

Among the 59 PRASEF isolates evaluated, 16 (27.1%) were resistant to imipenem and 58 (98.3%) to piperacillin, according to the results of the broth dilution reference method; however, no isolate was resistant to ampicillin or amoxicillin using the same reference method, indicating that ampicillin susceptibility should not be used to predict susceptibility of imipenem and piperacillin for *E. faecalis* with penicillin resistance phenotype.

As demonstrated in Table 1, the Etest revealed good accuracy (CA > 90%) for all beta-lactams, except for imipenem (CA of 85.1%), although no VME (false susceptibility) was observed for the latter drug. Notably, most imipenem false-resistant isolates (7 of 11) exhibited MIC values around the CLSI breakpoint. All beta-lactams presented good EA rates, except for piperacillin, which revealed a great variability in MIC values, particularly in the PRASEF isolates.

In contrast to Etest, the disk diffusion method presented good accuracy (CA > 90%) only for penicillin and amoxicillin. As illustrated in Fig. 1, the two PRASEF isolates erroneously detected as penicillin susceptible by the diffusion disk exhibited an MIC of 16 μg/mL (cutoff point), and one of them presented an inhibition halo of 15 mm (cutoff point). Notably, all *E. faecalis* isolates erroneously categorized as resistant to ampicillin (*n* = 13; 17.6%) and amoxicillin (*n* = 4; 5.4%) by disk diffusion were PRASEF isolates (Fig. 1). They exhibited MIC values ranging from 2 to 4 µg/mL for amoxicillin or from 2 to 8 µg/mL for ampicillin and inhibition halos of 15–16 mm, which are close to the cutoff point of 17 mm.

Imipenem presented poor performance in the disk diffusion method (CA, 55.4%). High rates of both VME (37.5%) and ME (46.5%) were found for this beta-lactam; however, notably, these false susceptible and false resistant isolates were PRASEF isolates (Fig. 1). A high rate of VME (24.15%) but low ME (6.3%) was observed for piperacillin, and the CA rate was unacceptable (79.7%).

As illustrated in Fig. 1, the MIC values for the beta-lactams evaluated tended to be higher among the PRASEF isolates in comparison to those of PSASEF. Moreover, PSASEF isolates usually displayed larger disk zone diameters than PRASEF. Presumably, the inhibition zones of most of the PRASEF isolates correspond exactly or are extremely close to the CLSI breakpoint values. This could lead to higher misinterpretation in the isolates observed from this group using the disk diffusion method, particularly for imipenem and piperacillin.

Only 16 of the 74 *E. faecalis* isolates susceptible to ampicillin were susceptible to piperacillin using the broth dilution method in this study. In another study, it was reported that ampicillin results could be extrapolated to piperacillin in 98.2% of *E. faecalis* analyzed using the same method; however, as the penicillin susceptibility was not determined in that study, presumably, the isolates evaluated were mainly PSASEF [15].

Interestingly, a fuzzy zone edge with a double halo of growth inhibition was observed in all beta-lactams tested by disk diffusion and Etest only for the PRASEF isolates. This needs to be further assessed because it could influence the reading and the interpretative criteria of beta-lactam susceptibility testing. As generally recommended by CLSI, the inner zone of complete growth inhibition, observed by the naked eye, was interpreted in the present study.

Although the present study provides valuable insights into the population of penicillin-resistant *E. faecalis* and the methods for testing beta-lactam susceptibilities, it has certain limitations. First, we did not truly test for ampicillin- or amoxicillin-resistant isolates as determined by the reference method; however, it should be emphasized that these isolates are rarely found worldwide, which precluded the assessment of VME rates for both beta-lactams. Second, only isolates from one tertiary care hospital were included, which might have resulted in a bias due to in-hospital clonal expansion of a few lineages. In contrast, we estimated that the clonal bias must be limited since our collection covers eight years and derives from different wards.

In conclusion, Etest is efficient in testing beta-lactam susceptibilities of PRASEF isolates, whereas the disk diffusion method revealed poor performance, mainly for imipenem and piperacillin. Therefore, a careful analysis of antimicrobial susceptibility tests for the emerging population of penicillin-resistant *E. faecalis* is warranted, particularly for predicting piperacillin or imipenem susceptibility using the ampicillin results. Thus, further studies are needed to explore different disk brands and PRASEF isolates from other institutions.

## Data Availability

The datasets used and analyzed during the present study are available from the corresponding author upon reasonable request.

## Abbreviations

CA: Categorical agreement
CLSI: Clinical and Laboratory Standards Institute
EA: Essential agreement
EUCAST: European Committee on Antimicrobial Susceptibility Testing
E. faecalis: Enterococcus faecalis
ME: Major error
MIC: Minimum inhibitory concentration
PRASEF: Penicillin-resistant, ampicillin-susceptible *Enterococcus faecalis*
PSASEF: Penicillin-susceptible, ampicillin-susceptible *E. faecalis*
VME: Very major error
